# Ethyl 4-(3-ethyl-5-oxo-4,5-dihydro-1*H*-1,2,4-triazol-4-yl)benzoate

**DOI:** 10.1107/S160053681001603X

**Published:** 2010-05-08

**Authors:** Yasemin Ünver, Yavuz Köysal, Hasan Tanak, Dilek Ünlüer, Şamil Işık

**Affiliations:** a Department of Chemistry, Karadeniz Technical University, TR-61080 Trabzon, Turkey; b Samsun Vocational School, Ondokuz Mayıs University, TR-55139 Samsun, Turkey; c Department of Physics, Ondokuz Mayıs University, TR-55139, Samsun, Turkey

## Abstract

In the title compound, C_13_H_15_N_3_O_3_, the dihedral angle between the two aromatic ring is 51.06 (1)°. In the crystal, mol­ecules are connected by pairs of N—H⋯O hydrogen bonds into centrosymmetric dimers.

## Related literature

For the pharmacological activity of 1,2,4-triazole compounds, see: Chiu & Huskey (1998 ▶); Eliott *et al.* (1986 ▶, 1987 ▶); Griffin & Mannion (1986 ▶, 1987, 1987 ▶); Heubach *et al.* (1975 ▶, 1979 ▶); Husain & Amir (1986 ▶, 1987 ▶,); Tanaka (1974 ▶, 1975 ▶); Tsukuda *et al.* (1998 ▶); Witkoaski *et al.* (1972 ▶). For the biological activity of the triazole family, see: Unver *et al.* (2008 ▶, 2009 ▶). For a related structure, see: Tanak *et al.* (2010 ▶).
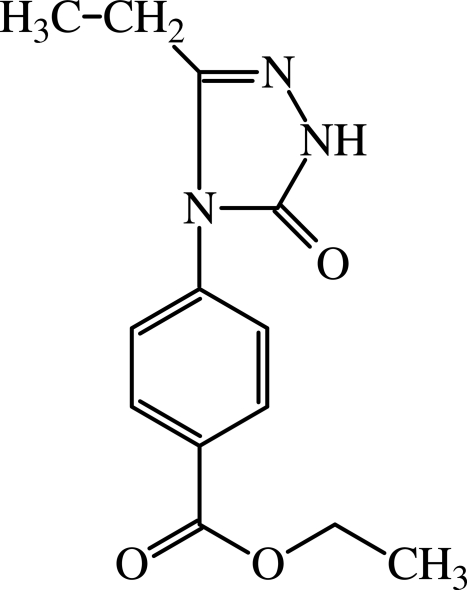


## Experimental

### 

#### Crystal data

C_13_H_15_N_3_O_3_
*M* *_r_* = 261.28
Monoclinic, 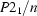

*a* = 13.6111 (11) Å
*b* = 4.0970 (2) Å
*c* = 24.172 (2) Å
β = 100.063 (7)°
*V* = 1327.20 (17) Å^3^
*Z* = 4
Mo *K*α radiation
μ = 0.10 mm^−1^
*T* = 293 K
0.80 × 0.41 × 0.13 mm

#### Data collection

Stoe IPDS 2 diffractometer
8189 measured reflections
2581 independent reflections
1606 reflections with *I* > 2σ(*I*)
*R*_int_ = 0.042

#### Refinement

*R*[*F* ^2^ > 2σ(*F* ^2^)] = 0.038
*wR*(*F* ^2^) = 0.095
*S* = 0.93
2581 reflections
216 parameters
H atoms treated by a mixture of independent and constrained refinement
Δρ_max_ = 0.13 e Å^−3^
Δρ_min_ = −0.14 e Å^−3^

### 

Data collection: *X-AREA* (Stoe & Cie, 2002 ▶); cell refinement: *X-AREA*; data reduction: *X-RED32* (Stoe & Cie, 2002 ▶); program(s) used to solve structure: *SHELXS97* (Sheldrick, 2008 ▶); program(s) used to refine structure: *SHELXL97* (Sheldrick, 2008 ▶); molecular graphics: *ORTEP-3 for Windows* (Farrugia, 1997 ▶); software used to prepare material for publication: *WinGX* (Farrugia, 1999 ▶).

## Supplementary Material

Crystal structure: contains datablocks I, global. DOI: 10.1107/S160053681001603X/bt5258sup1.cif

Structure factors: contains datablocks I. DOI: 10.1107/S160053681001603X/bt5258Isup2.hkl

Additional supplementary materials:  crystallographic information; 3D view; checkCIF report

## Figures and Tables

**Table 1:** Hydrogen-bond geometry (Å, °)

*D*—H⋯*A*	*D*—H	H⋯*A*	*D*⋯*A*	*D*—H⋯*A*
N1—H1⋯O1^i^	0.883 (19)	1.94 (2)	2.808 (2)	169.5 (18)

Symmetry code: (i) 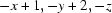.

**Fig. 1.**
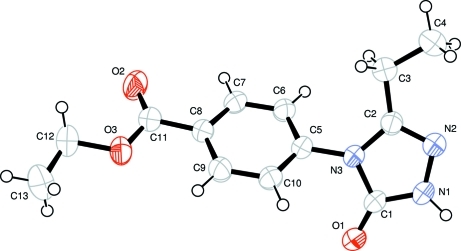
A view of the title compound with the atom-numbering scheme and 50% probability displacement ellipsoids.

**Fig. 2.**
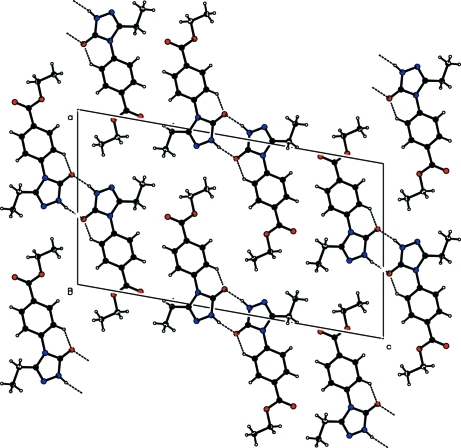
A partial packing diagram of the title compound.

## supplementary crystallographic information

### Comment

1,2,4-triazole compounds posses important pharmacology activities such as antifungal and antiviral activities. Examples of such compounds bearing the 1,2,4- triazole residues are fluconazole (Tsukuda *et al.*, 1998), the powerful azole antifungal agent as well as the potent antiviral N- nucleoside ribavirin (Witkoaski *et al.*, 1972). Furthermore, various 1,2,4-triazole derivatives have been reported as fungicidal (Heubach *et al.*, 1975, 1979), insecticidal (Tanaka, 1974, 1975), antimicrobial, (Griffin & Mannion, 1986, 1987) as well as anticonvulsants (Husain & Amir, 1986, 1987), antidepressants (Chiu & Huskey, 1998), and plant growth regulator anticoagulants (Eliott *et al.*, 1986, 1987). Our laboratories reported the some biological activity of the triazole family (Unver *et al.*,  2008; Unver *et al.*, 2009). It is known that 1,2,4-triazol moieties interact strongly with heme iron, and aromatic substituents on the triazoles are very effective for interacting with the active site of aromatase. Furthermore, It was reported that compounds having triazole moieties such as Vorozole, Anastrozole and Letrozole appear to be very effective aromatase inhibitors very useful for preventing breast cancer.

In the title compound, the plane of the -C(=O)—O- group is inclined at the angle of 4.23 (1)° with respect to the benzoate ring. The dihedral angle between the two aromatic ring is 51.06 (1)°. The 1,2,4-triazole ring is strictly planar and the maximum deviation of -0.0016 (2)Å for atom C1. The double bond distance in the triazol group is good agreement with our previous report,5-benzyl-4- (3,4-dimethoxyphenethyl)-2*H*-1,2,4-triazol-3(4*H*)-one (Tanak *et al.*, 2010).

The molecules are connected by intermolecular N—H···O hydrogen bonds to centrosymmetric dimers. generating eight-membered ring, producing a R_2_^2^(8) motif (Bernstein *et al.*, 1995).

### Experimental

Ethyl 2-(1-ethoxypropylidene)hydrazinecarboxylate (10 mmol) and ethyl 4-amino benzoate (10 mmol) was mixed without solvent and heated at 433-443°K for 2 h. The formed solid products were separated by filtration, purified by crystallization twice from ethanol, washed with Et_2_O ether and dried in a vacuum. m p: 446°K.

### Refinement

The H atoms of the phenyl ring  were positioned geometrically and refined using a riding model with C—H = 0.93 Å and U(H)=1.2U_eq_(C). The remaining H atoms were  freely refined.

### Crystal data

**Table:**

C_13_H_15_N_3_O_3_	*F*(000) = 552
*M**_r_* = 261.28	*D*_x_ = 1.308 Mg m^−^^3^
Monoclinic, *P*2_1_/*n*	Mo *K*α radiation, λ = 0.71073 Å
*a* = 13.6111 (11) Å	Cell parameters from 10833 reflections
*b* = 4.0970 (2) Å	θ = 1.5–27.2°
*c* = 24.172 (2) Å	µ = 0.10 mm^−^^1^
β = 100.063 (7)°	*T* = 293 K
*V* = 1327.20 (17) Å^3^	Prism, colourless
*Z* = 4	0.80 × 0.41 × 0.13 mm

### Data collection

**Table:**

Stoe IPDS 2 diffractometer	1606 reflections with *I* > 2σ(*I*)
Radiation source: fine-focus sealed tube	*R*_int_ = 0.042
graphite	θ_max_ = 26.0°, θ_min_ = 1.6°
Detector resolution: 6.67 pixels mm^-1^	*h* = −16→15
rotation method scans	*k* = −4→5
8189 measured reflections	*l* = −29→29
2581 independent reflections	

### Refinement

**Table:**

Refinement on *F*^2^	Primary atom site location: structure-invariant direct methods
Least-squares matrix: full	Secondary atom site location: difference Fourier map
*R*[*F*^2^ > 2σ(*F*^2^)] = 0.038	Hydrogen site location: inferred from neighbouring sites
*wR*(*F*^2^) = 0.095	H atoms treated by a mixture of independent and constrained refinement
*S* = 0.93	*w* = 1/[σ^2^(*F*_o_^2^) + (0.0488*P*)^2^] where *P* = (*F*_o_^2^ + 2*F*_c_^2^)/3
2581 reflections	(Δ/σ)_max_ < 0.001
216 parameters	Δρ_max_ = 0.13 e Å^−^^3^
0 restraints	Δρ_min_ = −0.13 e Å^−^^3^

### Special details

**Table:**

Geometry. All esds (except the esd in the dihedral angle between two l.s. planes) are estimated using the full covariance matrix. The cell esds are taken into account individually in the estimation of esds in distances, angles and torsion angles; correlations between esds in cell parameters are only used when they are defined by crystal symmetry. An approximate (isotropic) treatment of cell esds is used for estimating esds involving l.s. planes.
Refinement. Refinement of *F*^2^ against ALL reflections. The weighted *R*-factor wR and goodness of fit *S* are based on *F*^2^, conventional *R*-factors *R* are based on *F*, with *F* set to zero for negative *F*^2^. The threshold expression of *F*^2^ > σ(*F*^2^) is used only for calculating *R*-factors(gt) etc. and is not relevant to the choice of reflections for refinement. *R*-factors based on *F*^2^ are statistically about twice as large as those based on *F*, and *R*- factors based on ALL data will be even larger.

### Fractional atomic coordinates and isotropic or equivalent isotropic displacement parameters (Å^2^)

**Table:**

	*x*	*y*	*z*	*U*_iso_*/*U*_eq_	
C1	0.44143 (13)	0.7739 (5)	0.05726 (6)	0.0485 (5)	
C2	0.51671 (12)	0.5332 (4)	0.13682 (6)	0.0440 (4)	
C3	0.53173 (14)	0.3581 (6)	0.19150 (8)	0.0525 (5)	
C4	0.63819 (16)	0.2621 (7)	0.21203 (9)	0.0631 (6)	
C5	0.32736 (11)	0.5585 (5)	0.11912 (6)	0.0432 (4)	
C6	0.30553 (12)	0.6504 (5)	0.17095 (6)	0.0466 (5)	
H6	0.3534	0.7542	0.1973	0.056*	
C7	0.21229 (12)	0.5858 (5)	0.18282 (6)	0.0473 (4)	
H7	0.1975	0.6437	0.2177	0.057*	
C8	0.14040 (12)	0.4365 (5)	0.14379 (6)	0.0450 (4)	
C9	0.16301 (13)	0.3535 (5)	0.09164 (7)	0.0533 (5)	
H9	0.1146	0.2559	0.0648	0.064*	
C10	0.25609 (12)	0.4141 (5)	0.07938 (6)	0.0515 (5)	
H10	0.2708	0.3579	0.0445	0.062*	
C11	0.04218 (13)	0.3608 (5)	0.15961 (7)	0.0512 (5)	
C12	−0.11800 (16)	0.1271 (8)	0.13044 (10)	0.0724 (7)	
C13	−0.1753 (2)	−0.0096 (12)	0.07756 (15)	0.1015 (11)	
N1	0.54074 (11)	0.7686 (5)	0.06243 (6)	0.0562 (5)	
N2	0.58809 (10)	0.6223 (4)	0.11107 (5)	0.0519 (4)	
N3	0.42458 (9)	0.6181 (4)	0.10615 (5)	0.0447 (4)	
O1	0.37870 (9)	0.8859 (4)	0.01948 (5)	0.0624 (4)	
O2	0.02253 (9)	0.4134 (4)	0.20563 (5)	0.0726 (5)	
O3	−0.02131 (9)	0.2283 (4)	0.11801 (5)	0.0660 (4)	
H1	0.5731 (14)	0.864 (5)	0.0382 (8)	0.062 (6)*	
H3A	0.4910 (15)	0.170 (5)	0.1871 (8)	0.069 (6)*	
H3B	0.5059 (14)	0.489 (5)	0.2178 (8)	0.065 (6)*	
H4A	0.6436 (14)	0.140 (5)	0.2481 (9)	0.074 (6)*	
H4B	0.6795 (18)	0.443 (6)	0.2188 (9)	0.090 (8)*	
H4C	0.6648 (16)	0.115 (6)	0.1853 (10)	0.086 (7)*	
H12A	−0.1044 (16)	−0.029 (6)	0.1621 (9)	0.085 (7)*	
H12B	−0.1459 (17)	0.324 (6)	0.1422 (9)	0.083 (8)*	
H13A	−0.234 (2)	−0.096 (8)	0.0840 (12)	0.126 (10)*	
H13B	−0.138 (3)	−0.175 (9)	0.0604 (14)	0.153 (16)*	
H13C	−0.189 (2)	0.174 (8)	0.0528 (12)	0.120 (12)*	

### Atomic displacement parameters (Å^2^)

**Table:**

	*U*^11^	*U*^22^	*U*^33^	*U*^12^	*U*^13^	*U*^23^
C1	0.0429 (10)	0.0675 (13)	0.0373 (8)	−0.0030 (9)	0.0133 (7)	0.0024 (9)
C2	0.0395 (9)	0.0531 (12)	0.0399 (8)	−0.0052 (8)	0.0087 (7)	−0.0039 (8)
C3	0.0470 (11)	0.0639 (15)	0.0468 (10)	0.0000 (11)	0.0083 (8)	0.0064 (10)
C4	0.0521 (12)	0.0768 (17)	0.0572 (12)	0.0002 (13)	0.0006 (10)	0.0093 (12)
C5	0.0374 (9)	0.0534 (12)	0.0404 (8)	−0.0005 (8)	0.0111 (7)	0.0043 (8)
C6	0.0433 (9)	0.0591 (13)	0.0377 (8)	−0.0077 (9)	0.0083 (7)	−0.0033 (8)
C7	0.0462 (9)	0.0612 (12)	0.0364 (8)	−0.0028 (9)	0.0127 (7)	−0.0011 (8)
C8	0.0377 (9)	0.0572 (12)	0.0409 (8)	0.0010 (8)	0.0095 (7)	0.0033 (8)
C9	0.0431 (10)	0.0739 (14)	0.0428 (9)	−0.0079 (9)	0.0074 (7)	−0.0078 (9)
C10	0.0449 (10)	0.0743 (13)	0.0370 (8)	−0.0026 (10)	0.0118 (7)	−0.0052 (9)
C11	0.0417 (10)	0.0646 (14)	0.0481 (9)	−0.0004 (9)	0.0095 (8)	0.0047 (9)
C12	0.0435 (12)	0.096 (2)	0.0784 (15)	−0.0156 (13)	0.0131 (11)	0.0078 (15)
C13	0.0636 (17)	0.135 (3)	0.098 (2)	−0.039 (2)	−0.0063 (16)	0.010 (2)
N1	0.0414 (9)	0.0862 (13)	0.0438 (8)	−0.0034 (8)	0.0151 (6)	0.0120 (8)
N2	0.0424 (8)	0.0710 (11)	0.0430 (7)	−0.0024 (8)	0.0095 (6)	0.0047 (7)
N3	0.0375 (8)	0.0609 (10)	0.0370 (7)	−0.0037 (7)	0.0102 (6)	0.0038 (7)
O1	0.0463 (7)	0.0976 (11)	0.0452 (6)	0.0052 (7)	0.0137 (6)	0.0196 (7)
O2	0.0552 (8)	0.1122 (13)	0.0562 (7)	−0.0142 (8)	0.0256 (6)	−0.0088 (8)
O3	0.0414 (7)	0.1015 (12)	0.0556 (7)	−0.0182 (7)	0.0100 (6)	−0.0041 (7)

### Geometric parameters (Å, °)

**Table:**

C1—O1	1.2251 (19)	C7—H7	0.9300
C1—N1	1.336 (2)	C8—C9	1.391 (2)
C1—N3	1.397 (2)	C8—C11	1.486 (2)
C2—N2	1.295 (2)	C9—C10	1.373 (2)
C2—N3	1.385 (2)	C9—H9	0.9300
C2—C3	1.486 (2)	C10—H10	0.9300
C3—C4	1.500 (3)	C11—O2	1.2080 (19)
C3—H3A	0.94 (2)	C11—O3	1.322 (2)
C3—H3B	0.95 (2)	C12—O3	1.460 (2)
C4—H4A	1.00 (2)	C12—C13	1.486 (4)
C4—H4B	0.93 (3)	C12—H12A	0.99 (2)
C4—H4C	1.00 (2)	C12—H12B	0.96 (2)
C5—C10	1.374 (2)	C13—H13A	0.92 (3)
C5—C6	1.389 (2)	C13—H13B	0.98 (4)
C5—N3	1.433 (2)	C13—H13C	0.96 (3)
C6—C7	1.375 (2)	N1—N2	1.376 (2)
C6—H6	0.9300	N1—H1	0.883 (19)
C7—C8	1.378 (2)		
			
O1—C1—N1	129.64 (15)	C10—C9—C8	120.66 (16)
O1—C1—N3	127.27 (15)	C10—C9—H9	119.7
N1—C1—N3	103.09 (14)	C8—C9—H9	119.7
N2—C2—N3	110.93 (14)	C9—C10—C5	119.49 (15)
N2—C2—C3	124.44 (15)	C9—C10—H10	120.3
N3—C2—C3	124.63 (14)	C5—C10—H10	120.3
C2—C3—C4	113.30 (17)	O2—C11—O3	123.57 (16)
C2—C3—H3A	107.9 (12)	O2—C11—C8	123.62 (16)
C4—C3—H3A	109.7 (12)	O3—C11—C8	112.81 (14)
C2—C3—H3B	108.3 (12)	O3—C12—C13	106.6 (2)
C4—C3—H3B	112.2 (11)	O3—C12—H12A	106.8 (13)
H3A—C3—H3B	105.0 (17)	C13—C12—H12A	114.8 (13)
C3—C4—H4A	109.9 (12)	O3—C12—H12B	103.8 (14)
C3—C4—H4B	111.7 (15)	C13—C12—H12B	113.4 (14)
H4A—C4—H4B	107.3 (17)	H12A—C12—H12B	110 (2)
C3—C4—H4C	112.6 (13)	C12—C13—H13A	110.1 (18)
H4A—C4—H4C	106.5 (18)	C12—C13—H13B	113 (2)
H4B—C4—H4C	109 (2)	H13A—C13—H13B	110 (3)
C10—C5—C6	120.72 (15)	C12—C13—H13C	104.8 (18)
C10—C5—N3	119.11 (14)	H13A—C13—H13C	109 (2)
C6—C5—N3	120.16 (14)	H13B—C13—H13C	110 (3)
C7—C6—C5	119.14 (15)	C1—N1—N2	113.73 (14)
C7—C6—H6	120.4	C1—N1—H1	123.0 (12)
C5—C6—H6	120.4	N2—N1—H1	123.1 (12)
C6—C7—C8	120.88 (15)	C2—N2—N1	104.77 (13)
C6—C7—H7	119.6	C2—N3—C1	107.48 (13)
C8—C7—H7	119.6	C2—N3—C5	128.63 (13)
C7—C8—C9	119.07 (15)	C1—N3—C5	123.87 (13)
C7—C8—C11	118.65 (14)	C11—O3—C12	116.92 (15)
C9—C8—C11	122.26 (15)		
			
N2—C2—C3—C4	−5.8 (3)	N3—C2—N2—N1	0.0 (2)
N3—C2—C3—C4	173.1 (2)	C3—C2—N2—N1	178.99 (19)
C10—C5—C6—C7	2.0 (3)	C1—N1—N2—C2	0.2 (2)
N3—C5—C6—C7	−178.93 (17)	N2—C2—N3—C1	−0.1 (2)
C5—C6—C7—C8	−1.0 (3)	C3—C2—N3—C1	−179.15 (19)
C6—C7—C8—C9	−0.6 (3)	N2—C2—N3—C5	178.63 (17)
C6—C7—C8—C11	177.66 (18)	C3—C2—N3—C5	−0.4 (3)
C7—C8—C9—C10	1.1 (3)	O1—C1—N3—C2	−179.77 (19)
C11—C8—C9—C10	−177.07 (19)	N1—C1—N3—C2	0.23 (19)
C8—C9—C10—C5	−0.1 (3)	O1—C1—N3—C5	1.4 (3)
C6—C5—C10—C9	−1.5 (3)	N1—C1—N3—C5	−178.60 (16)
N3—C5—C10—C9	179.42 (17)	C10—C5—N3—C2	−128.50 (19)
C7—C8—C11—O2	−2.5 (3)	C6—C5—N3—C2	52.4 (3)
C9—C8—C11—O2	175.7 (2)	C10—C5—N3—C1	50.1 (3)
C7—C8—C11—O3	177.81 (17)	C6—C5—N3—C1	−129.01 (19)
C9—C8—C11—O3	−4.0 (3)	O2—C11—O3—C12	−3.3 (3)
O1—C1—N1—N2	179.7 (2)	C8—C11—O3—C12	176.4 (2)
N3—C1—N1—N2	−0.3 (2)	C13—C12—O3—C11	179.5 (3)

### Hydrogen-bond geometry (Å, °)

**Table:**

*D*—H···*A*	*D*—H	H···*A*	*D*···*A*	*D*—H···*A*
N1—H1···O1^i^	0.883 (19)	1.94 (2)	2.808 (2)	169.5 (18)

Symmetry codes: (i) −*x*+1, −*y*+2, −*z*.
